# Giant intracholecystic papillary tubular adenoma of the gall bladder with gall stones in an elderly woman; case report

**DOI:** 10.1016/j.amsu.2019.09.006

**Published:** 2019-09-19

**Authors:** Ayad Ahmad Mohammed

**Affiliations:** Department of Surgery, College of Medicine, University of Duhok, Kurdistan Region, Iraq; Azadi Teaching Hospital, 8 Nakhoshkhana Road, 1014 AM, Duhok City, DUHOK, Kurdistan Region, Iraq

**Keywords:** Gall bladder polys, Papillary tubular adenoma, Gall stones, Cholecystectomy

## Abstract

Gall bladder polyps occur in 0.4% of patients undergoing cholecystectomy, the majority of gall bladder polyps are benign, they are classified into 3 types: epithelial or adenomatous polyps, mesenchymal polyps, and pseudopolyps. Gall bladder polys mostly affect females and those more than 50 years of age. Ultrasound is a very sensitive tool in the diagnosis.

An 88-year-old woman presented with epigastric pain and right hypochondrial pain, fever, and vomiting for 1 week. Clinical examination showed jaundice and tenderness at the right hypochondrial region. Investigations showed elevated WBC, bilirubin level and the alkaline phosphatase. MRCP showed multiple gall stones with a large irregular polyp in the fundus of the gall bladder, and dilated common bile duct with multiple stones in the lumen of common bile duct. Cholecystectomy was done with exploration of the common bile duct with extraction of stones, T-tube was placed inside the CBD. At the 14th day T-tube cholangiography was done which showed passage of the dye to the duodenum, the tube was extracted and the patient was discharged home with no postoperative complications. The histopathology showed intracholecystic papillary tubular adenoma of the gall bladder with no evidence of malignancy.

The general indications of surgery for gall bladder polyps include the size if more than 10 mm especially if solitary, the presence of associated gall stones, the age if more than 60 years, and if the polyps are causing symptoms. In this patients the large size of the polyp and obstructive jaundice were the two indications for surgery.

## Highlights

• Gall bladder polyps are commonly diagnosed and may be associated with gall stones.
• Most patients are worried about the possibility of malignancy.
• Laparoscopic cholecystectomy can be safely done when there is indication for surgery.

## 1 Introduction

Gall bladder polys are defined as any elevated lesion above its mucosal surface. It is estimated that polyps occur in 0.4% of patients undergoing cholecystectomy, polyps can be diagnosed in up to 7% of patients before cholecystectomy for gall stones, but the majority are small and diagnosed after cholecystectomy [1]

**Fig. 1:**
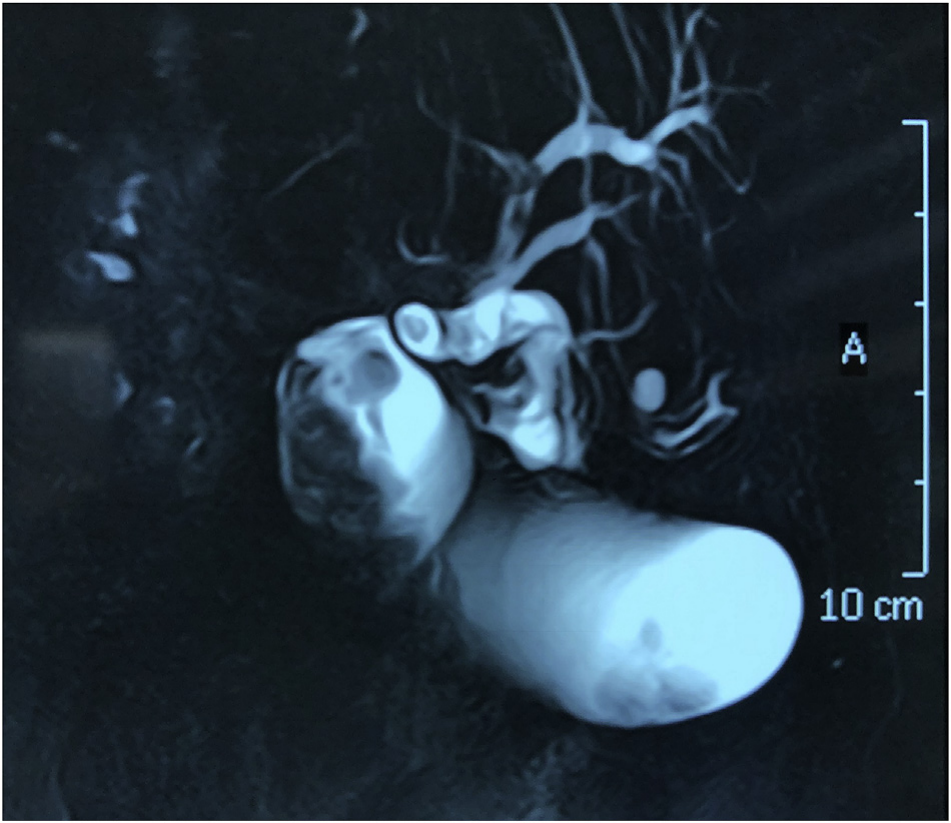
MRCP picture showing a large polyp at the fundus of the gall bladder, the dilated common bile duct and the presence of multiple stones in the lumen of the gall bladder and the common bile duct.

**Fig. 2:**
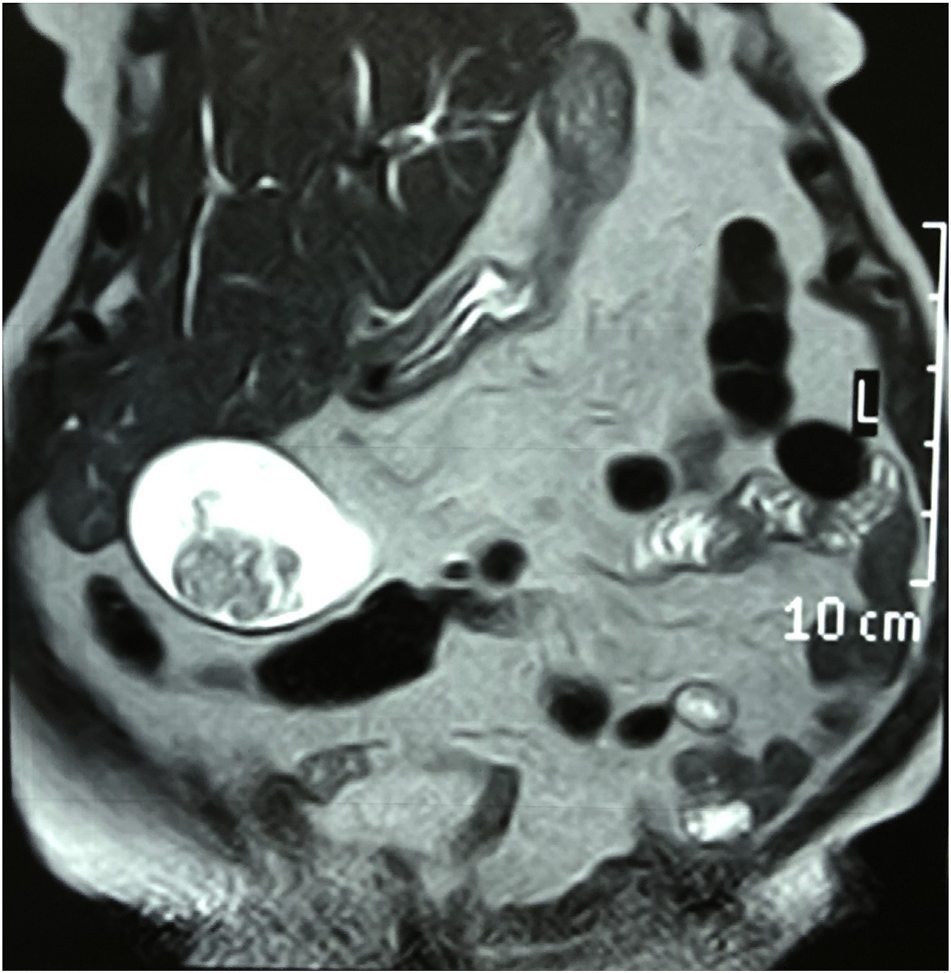
A T-2 weighted MRI picture showing a poly inside the lumen of the gall bladder.

**Fig. 3:**
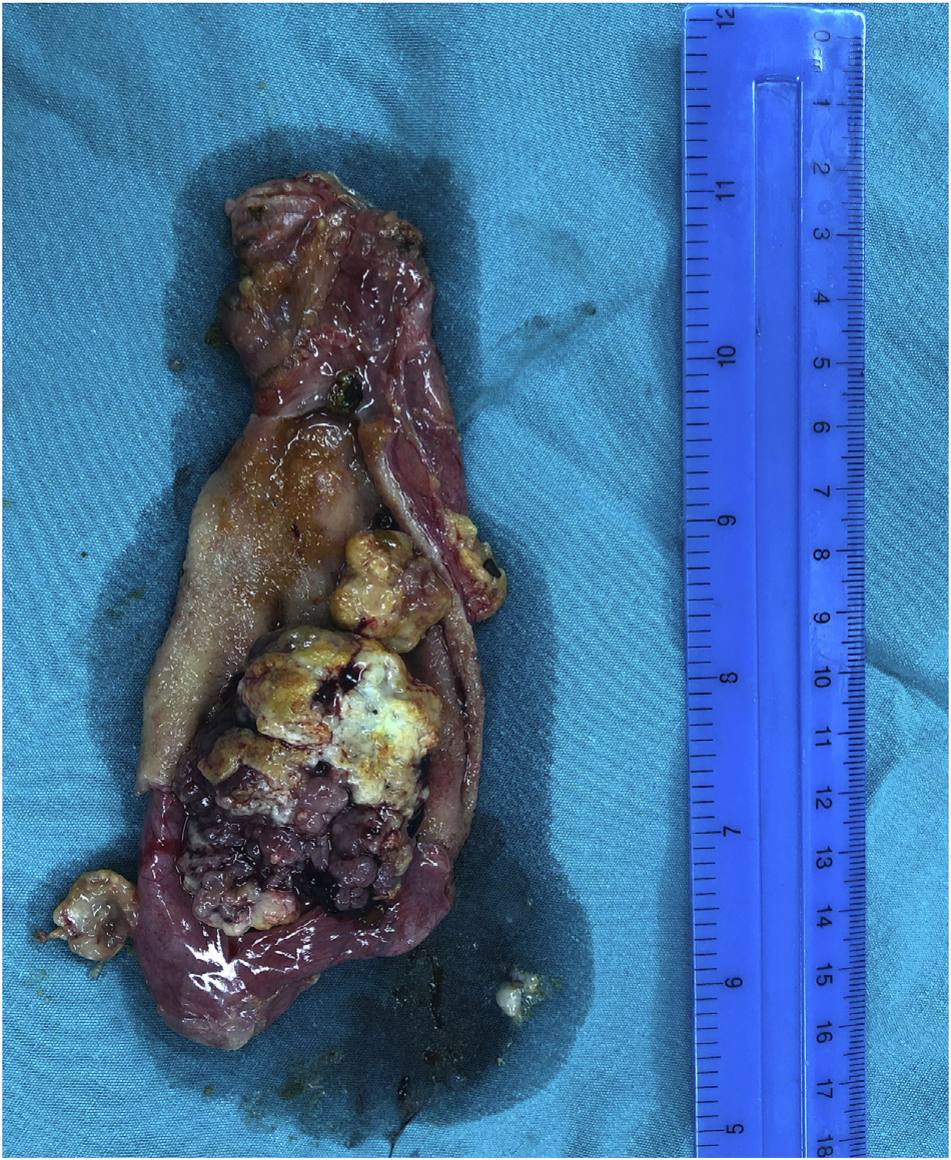
An intraoperative picture showing a large 5 cm polyp with irregular surface arising from the wall of the gall bladder.

**Fig. 4:**
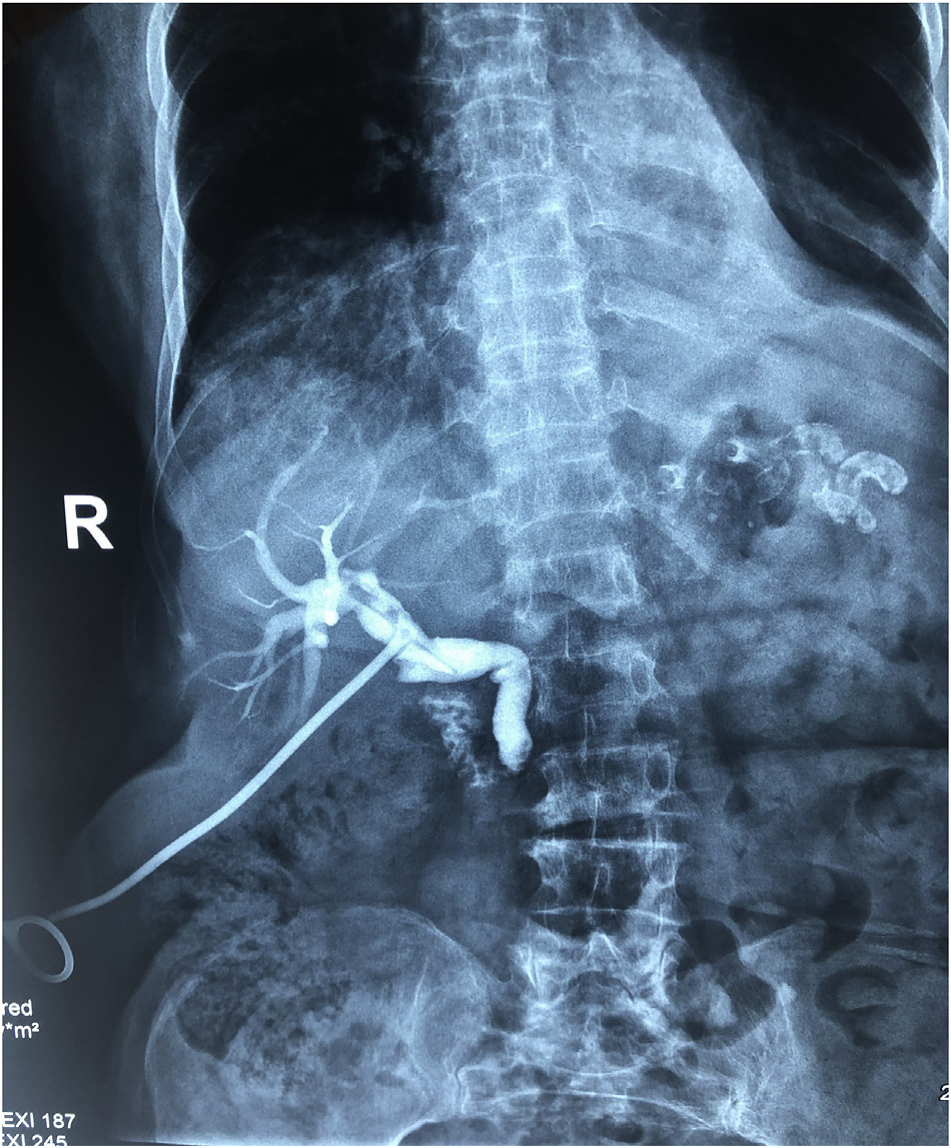
Postoperative T-tube cholangiography showing the passage of the contrast to the lumen of the duodenum with no residual stones in the lumen of the common bile duct.

The majority of gall bladder polyps are benign lesions that may be discovered incidentally or in association with gall stones. There are many pathological types of gall bladder polyps such as epithelial or adenomatous polyps, mesenchymal polyps, and pseudopolyps. Polyps may be primary occurring de novo or secondary association with metachromatic leukodystrophy, Peutz-Jeghers syndrome, inflammatory bowel diseases, primary sclerosing cholangitis or pancreato-biliary malunion [[2], [3], [4]].

Most cases of gall bladder polys are females, more than 50 years of age and the majority are detected incidentally. Once gall bladder polyps are diagnosed most patients are concerned whether these lesions are benign or malignant and the possibility of their malignant transformation [1,5].

Ultrasound of the gall bladder is a very sensitive tool in diagnosing gall bladder polyps, polys are differentiated from gall stones by their constant position despite changing the position of the patient during ultrasound examination [6].

The recommended follow up for gall bladder polyps is to perform ultrasound every 3–6 months for the first 2 years, then frequent follow up is probably unnecessary [7].

The work in this case report has been reported in line with the SCARE 2018 criteria [8].

## 2 Patient information

An 88-year-old woman was presented with epigastric pain and right hypochondrial pain, fever, and vomiting for 1 week. She was admitted to the surgical unit.

The patient had history of hypertension and she was on regular medical therapy.

**Clinical findings**: During clinical examination the pulse rate was 95 b/m, the BP was 170/95 mmHg, and the temperature was 38.3, she had jaundice and the adnominal examination showed a scar of previous appendicectomy which was done before several years and tenderness at the right hypochondrial region.

**Diagnostic Assessment**: Investigations showed elevated white blood cell count 14000 cmm, bilirubin level was 2.3 mg/dl and elevated alkaline phosphatase 842 IU/L. Ultrasound and magnetic resonance cholangiopancreatography showed evidence of multiple gall stones with a large irregular polyp in the region of the fundus of the gall bladder, and dilated common bile duct with evidence of multiple stones in the lumen of common bile duct. Figs. 1 and 2

**Therapeutic Intervention**: The patient was admitted to the hospital and she received parenteral antibiotics and hydration.

Surgery was done through right subcostal incision, cholecystectomy was performed with exploration of the common bile duct and extraction of all the stones from its lumen, T-tube was placed inside the CBD. Fig. 3.

The operation was done by a specialist general surgeon who has the experience in performing biliary surgeries. The family history was negative for malignant diseases and the patient had dementia diagnosed before 5 years.

**Follow-up and outcome**: The patient was admitted to the hospital for 1 week, at the 14th day T-tube cholangiography was done which showed the passage of the dye to the duodenal lumen with no residual stones, the T-tube was extracted and the patient was discharged home with no postoperative complications. Fig. 4.

The result of the histopathology showed dysplastic biliary epithelium arranged in tubular and papillary pattern, with no evidence of malignancy.

No specific post intervention considerations were undertaken.

## 3 Discussion

Most histopathological samples of gall bladder polys show small in size polyps due to the early presentation of most cases and the early surgical intervention by cholecystectomy for this reason cases of very large polyps are rarely reported nowadays [5].

The pathological classification of the gall bladder polyps depends on the tissue of origin, epithelial polyps or called adenomatous polyps are divided into 3 types: tubular polyps, papillary polyps or mixed type when contain both elements, mesenchymal polyps are further divided into: leiomyomas, hemangiomas, and lipomas, and pseudo-polyps which are further divided into: cholesterol polyps, inflammatory polyps, adenomatous polyps, and adenomyomatous polyps [4].

The indications of surgery for gall bladder polyps include the size of the polyp when exceeding 10 mm, solitary polyp, the presence of gall stones with the polyps, the advanced age of the patient when more than 60 years, as these factors are also associated with increased risk of gall bladder cancer. When the clinical symptoms are attributed to these polyps this may be another indication for surgery, polyps near the cystic duct may be the cause of impaired emptying of the gall bladder and hence may cause upper abdominal pain [[5], [6], [7],^9^].

The greatest concern is when the patients have some of the dyspeptic symptoms, many patients attribute their symptoms to theses polyps and the patients become very aware for the risk of malignant transformation, the problem of patient selection for surgery remains a great issue and many debate is still present between the authors worldwide [7].

Laparoscopic cholecystectomy is one of the most widely performed procedure all over the world, such operation can be done safely for patients when there is a surgical intervention for cholecystectomy [10].

**Patient Perspective:** The patient was unaware of her complain because she had dementia but the family were concerned about the possibility of malignancy before surgery.

**Informed Consent:** An informed written consent was taken from family for reporting this case and the accompanying images.

**Provenance and peer review:** Not commissioned, externally peer reviewed.

## Ethical approval

No ethical committee approval was needed; consent have been taken from the family to report their findings.

## Sources of funding

No source of funding other than the authors.

## Author contribution

The surgeon who performed the procedure: Dr Ayad Ahmad Mohammed.

Study design, writing, and the final approval of the manuscript: Dr Ayad Ahmad Mohammed.

## Conflicts of interest

No conflicts of interest present.

## Trial registry number

N/A.

## Guarantor

Dr Ayad Ahmad Mohammed.

## References

[1] Farinon A.M., Pacella A., Cetta F., Sianesi M. (1991). Adenomatous polyps of the gallbladder adenomas of the gallbladder. HPB Surg..

[2] Carrera G., Ochsner S.F. (1958). Polypoid mucosal lesions of gallbladder. J. Am. Med. Assoc..

[3] Stringer M.D., Ceylan H., Ward K., Wyatt J.I. (2003). Gallbladder polyps in children—classification and management. J. Pediatr. Surg..

[4] Csendes A., Burgos A.M., Csendes P., Smok G., Rojas J. (2001). Late follow-up of polypoid lesions of the gallbladder smaller than 10 mm. Ann. Surg..

[5] Adsay V., Jang K.-T., Roa J.C., Dursun N., Ohike N., Bagci P. (2012). Intracholecystic papillary-tubular neoplasms (ICPN) of the gallbladder (neoplastic polyps, adenomas, and papillary neoplasms that are≥ 1.0 cm): clinicopathologic and immunohistochemical analysis of 123 cases. Am. J. Surg. Pathol..

[6] Yang H., Sun Y., Wang Z. (1992). Polypoid lesions of the gallbladder: diagnosis and indications for surgery. Br. J. Surg..

[7] Lee K.F., Wong J., Li J.C.M., San Lai P.B. (2004). Polypoid lesions of the gallbladder. Am. J. Surg..

[8] Agha R.A., Borrelli M.R., Farwana R., Koshy K., Fowler A.J., Orgill D.P. (2018). The SCARE 2018 statement: updating consensus Surgical CAse REport (SCARE) guidelines. Int. J. Surg..

[9] Arif S.H., Hussein I.S., Mohammed A.A. (2019). Duplicated gall bladder with gall bladder polyp presenting with cholecystitis; case report with literature review. Int. J. Surg. Case Rep..

[10] Mohammed A.A., Arif S.H. (2019). Midline gallbladder makes a challenge for surgeons during laparoscopic cholecystectomy; case series of 6 patients. Ann. Med. Surg..

